# Comparing two methods of education (virtual versus traditional) on learning of Iranian dental students: a post-test only design study

**DOI:** 10.1186/1472-6920-14-45

**Published:** 2014-03-05

**Authors:** Fariborz Moazami, Ehsan Bahrampour, Mohammad Reza Azar, Farzad Jahedi, Marzieh Moattari

**Affiliations:** 1 Department of Endodontics, School of Dentistry, Shiraz University of Medical Sciences, Shiraz, Iran; 2 Oral and Maxillofacial Radiologist, Department of Oral and Maxillofacial Radiology, School of Dentistry, Shiraz University of Medical Sciences, Shiraz, Iran; 3 Department of Biomedical Informatics, Shiraz University of Medical Sciences, Shiraz, Iran; 4 Faculty of Nursing and Midwifery, Shiraz University of Medical Sciences, Shiraz, Iran

## Abstract

**Background:**

The importance of using technologies such as e-learning in different disciplines is discussed in the literature. Researchers have measured the effectiveness of e-learning in a number of fields.

Considering the lack of research on the effectiveness of online learning in dental education particularly in Iran, the advantages of these learning methods and the positive university atmosphere regarding the use of online learning. This study, therefore, aims to compare the effects of two methods of teaching (virtual versus traditional) on student learning.

**Methods:**

This post-test only design study approached 40, fifth year dental students of Shiraz University of Medical Sciences. From this group, 35 students agreed to participate. These students were randomly allocated into two groups, experimental (virtual learning) and comparison (traditional learning). To ensure similarity between groups, we compared GPAs of all participants by the Mann–Whitney U test (P > 0.05). The experimental group received a virtual learning environment courseware package specifically designed for this study, whereas the control group received the same module structured in a traditional lecture form. The virtual learning environment consisted of online and offline materials. Two identical valid, reliable post-tests that consisted of 40 multiple choice questions (MCQs) and 4 essay questions were administered immediately (15 min) after the last session and two months later to assess for knowledge retention. Data were analyzed by SPSS version 20.

**Results:**

A comparison of the mean knowledge score of both groups showed that virtual learning was more effective than traditional learning (effect size = 0.69).

**Conclusion:**

The newly designed virtual learning package is feasible and will result in more effective learning in comparison with lecture-based training. However further studies are needed to generalize the findings of this study.

## Background

Currently, the use of computers is increasing in dental education [1]. Electronic education has moved away from the hard medium to an online environment due to increased internet speed, accessibility and connectivity [2]. In a review, medical students have stated they prefer web tutorials rather than traditional lecture-based classes. Advantages such as accessibility, ease of use, freedom of navigation, high quality medical images and the possibility of repeat practice are among the reasons mentioned for their preference. Web-based learning is an important tool in evidence-based medicine because it is continually being developed and updated [3].

Despite its advantages, the primary drawbacks to online learning are technical issues and student isolation. Some students miss the interaction in a regular classroom, whereas self-directed learners are more successful in online education [4]. These findings may be the reason that integration of electronic education with classroom problem solving sessions or other activities is necessary [5-7].

Interactivity of web-based learning or internet-based e-learning in addition to providing access to existing learning modules, regardless of time and location, has enabled numerous researchers to use different learning management systems such as ILIAS as an open source system [8] or an interactive digital tool [9].

Different methods and designs have been applied to evaluate web-based learning. Grimes, in a qualitative study entitled “Student Perceptions of an Online Dental Terminology Course”, has described and analyzed the experiences of participants to determine their satisfaction level with online education. Study participants identified four themes - convenience, technical issues, sense of belonging, and learning style [4]. Questionnaires used in a study conducted at the University Of Birmingham School Of Dentistry identified the opinions and expectations of web-based courseware as a supplement to traditional teaching. They concluded that web-based courseware was a useful, additional resource for students [10].

These studies have primarily been conducted in Western countries. There is limited research that examines the effect of e-learning in Iran. Considering the gaps in the literature, cost-effectiveness of these programs and the university’s vision to develop international programs by expansion of virtual learning, therefore this study investigates the effects of virtual learning on “Rotary Instrumentation of Root Canals” among fifth year dental students at Shiraz University of Medical Sciences.

## Methods

### Study design

The study originated from need to compare knowledge acquisition and its retention between two groups of students. We used post-tests to analyze knowledge acquisition and its retention among students who, prior to the study, had not attended this course. We randomly assigned eligible fifth year dental students to the following two groups, experimental (virtual learning) and comparison (traditional learning).

The same lecturer used two different learning models (virtual and traditional) to plan a course on the topic “rotary instrumentation of root canals”. The study groups completed their courses over three consecutive weeks. Their improvement was assessed immediately and two months after completion of the course by a valid, reliable test.

### Participants

• **Selection criteria**

• Participants of the study were fifth year dental students. All had passed a total of 180 credits. Students had completed the majority of theoretical and practical courses.

• **Method for selection of participants (sampling)**

• We officially invited 45 students to voluntarily participate. Of these, 40 students agreed to participate and were randomly assigned to either the experimental or control groups. We used the Mann–Whitney U test to measure grade point averages (GPA) to ensure similarity among participants. There was no significant difference between the GPAs of both groups (P > 0.05). Two students in the experimental group were injured in an accident on the starting day of the course and were unable to participate. Three other students from the experimental group stated their unwillingness to continue participation in virtual classes. They were invited to attend the control group class, but excluded from the study. Ultimately 35 students completed the entire course, including examinations.

The control group (n = 20) attended a traditional lecture designed coursework while the experimental group (n = 15) participated in virtual learning of the same course. Both groups successfully completed the courses and participated in the examinations.

• **Recruitment of the subjects**

• As the attendance was voluntary, we made some interventions to increase the likelihood that students would participate. Interventions included selection of the subject according to the student’s felt needs. We held a briefing session to provide students with the necessary information regarding their attendance. Students were reassured that the test results would not be used in their evaluations.

The study necessitated that students have the ability to use a computer and the internet, [11] therefore we assessed students’ competencies by a form that consisted of relevant questions. This assessment ensured each student’s ability in using the program.

### Data collection

We controlled for variables by the following approaches:

1. Educational level was set only for dental school students in their fifth year.

2. Educational abilities were controlled by statistical analysis of students’ latest GPA.

3. To provide the same level of IT tools for learning, we required all experimental group participants to attend a training session on basic IT requirements and the e-learning application interface.

4. No participants had previous exposure to the course.

5. The same lecturer planned and presented both courses.

6. Both groups of students completed the courses in the same time frame.

7. Both courses had the same content comprised of the same teaching movies and presentation.

For both groups, the basic variable taken into consideration was the method of teaching, conventional versus virtual education. Knowledge acquisition and retention were examined according to students’ scores in the designed post-test.

### Educational methods

There were two methods in this study, conventional and virtual. In both, the learning objectives were designed by the same professor of endodontics who cited two common, important dental reference books.

• **Conventional method**

• The conventional method was a lecture-based course that included three, one hour sessions taught by a Professor of Endodontics. In this course, the sequence of PowerPoint slides and video were shown in the same order as the virtual learning method.

• **Virtual learning method**

• All of the learning objectives were designed the same as the conventional method, with the exception of the teaching method. We designed a virtual learning environment that included all corresponding flexibilities and user friendliness enriched with multimedia, as well as interactions among peers and the lecturer.

The sharable content object reference (SCORM) compliant LMS was equipped with online videoconference ability and a reporting system to follow students’ progress. Students in the experimental group were given a username and a password to login and the available options included the following: learning path (lessons), video conference environment, quiz and assignments, and contact with teacher and/or students.

Learning path (lessons) comprised a complete module that included the presentation and corresponding movies. This was designed such that users could not skip any slide, rather they were required to view the slides in order and time of the contents.

The video conference environment consisted of three online sessions scheduled for all attending participants.

Participants were required to complete two assignments. For each session, one online multiple choice question (MCQ) quiz was given to participants. Both activities were completed by feedback to participants in order to enable them to effectively understand the context.

All experimental group participants had access to a list of other participants and an online environment for sharing their ideas, either with or without the presence of the teacher through texts.

In addition to a training session, we developed a guideline for students to facilitate their use of e-learning. Using the guideline, students could easily learn how to access the website, login, submit their assignments, take the quizzes, and contact their teacher.

### Method of assessment

A post-test that included MCQ and essay questions was designed according to the lesson plan. Validity of the test was confirmed by a panel of endodontists. Its reliability was assessed by KR20 (R = 0.74). We evaluated all participants upon completion of the courses and two months later. The latter was for measurement of students’ knowledge retention. For each MCQ and essay question, we assigned a point value of 0.5 and 2.5, respectively for a total possible score of 30 points.

### Statistical analysis

Statistical analysis was performed by SPSS version 20. The Mann–Whitney U non-parametric test was used to compare participants’ GPAs. The effect size was measured to describe the magnitude of between group differences of the scores in the first and second post-tests.

### Ethical considerations

Students participated voluntarily after official invitation. Participants were assured that test results were confidential and did not constitute a part of their formal assessment. GPAs were calculated according to student identification numbers and were anonymous.

After the second post-test, all students in the control group received a copy of a compact disc that included all resources which were available to the experimental group.

This study was approved by the Ethics Committee of Shiraz University of Medical Sciences (ECSUMS).

### Study challenges

The experimental group was supposed to connect to the LMS from any location that had available internet access. However, participation in videoconference sessions was impossible from the home/dormitory environment due to a low speed internet connection. Thus, we used the intranet instead of the internet. We equipped 15 computers in different departments with microphones and webcams, then requested students to attend in sessions using the intranet. Other features of the LMS such as assignments, access to learning paths, quizzes, contact with the teacher and other students were accessed by students independent of time and location. Students also received a compact disc that contained the learning path of each session. Another challenge we encountered was an intranet disconnection in one of the videoconference sessions which was rescheduled at the next appropriate time.

## Results

This study enrolled 35, fifth year undergraduate dental students from the School of Dentistry, Shiraz University of Medical Sciences. Students’ GPA was 16.01. The mean GPA in the experimental (15.6 ± 0.86) and control (16.29 ± 1.05) groups did not significantly differ (Absolute difference = 0.69, P = 0.112).

The magnitude of the difference as measured by effect size was 0.66 for the first post-test and 0.59 for the second post-test (Table 1).

**Table 1:** Results of first and second post-test knowledge score from both study groups

**Study groups**	**First post-test Mean ± SD**	**Second post-test Mean ± SD**
**Control group (n = 20)**	19.25 ± 5.11	17.26 ± 3.35
**Experimental group (n = 15)**	22.45 ± 4.41	19.65 ± 4.88
**Absolute difference**	−3.20	−2.39
**Effect size**	0.66	0.59

## Discussion

The findings showed a favorable magnitude of difference for both post-tests in favor of virtual learning. This result was a valuable outcome because at the time of conducting this research our students had no previous experience with virtual learning.

We designed, implemented and evaluated the virtual learning method. Our experience in designing this method could be regarded as the starting point for the use of a virtual learning program in Iranian dental schools.

During implementation of the virtual learning program we encountered an intranet disconnection in one of the videoconference sessions. This session was scheduled for the next appropriate time. It appeared that the net disconnection did not interfere with students’ learning. Clark, in a review, stated that technologic problems did not interfere with the learning process. He mentioned that the teleconference-based and web-based delivery of educational content could be as effective as traditional classroom-based teaching [12]. In support, computer-managed instruction has been proposed by educators as an alternative teaching method to traditional classroom teaching without the sacrifice of quality education for certain courses [13]. Of note, it is believed that the development of such multimedia information and communication systems demands cooperative working teams of professionals who are able to master several areas of medical (dental) knowledge as well as the presentation of using different multimedia facilities [14].

We evaluated the effectiveness of this program by the administration of two post-tests. Although the subject of learning was practical-based we focused on cognitive, but not skill or performance acquisition. Studies have shown the effectiveness of a web-based course for skill development [3]. We considered students’ knowledge acquisition and knowledge retention by administering the same exam with a two-month interval. The results of the second post-test administered after two months showed similar knowledge retention when compared with the results of the first post-test. This result has supported the assumption that content provided by virtual and electronic learning can be better learned and retrieved.

Of note, the same teacher presented the lecture and online course. This might have impacted the manner in which the traditional lecture-based instruction was provided because the instructor was possibly influenced by the development of the virtual learning material. This instructor might have incorporated the online learning structure into the lecture-based instruction. The possible effect of the use of the same slides on the quality of lectures should not be undermined.

In the current study, the examination consisted of a combination of MCQs and essays to evaluate students’ knowledge. Written tests, if developed appropriately, measure higher order thinking. However if later stages of cognitive measurement such as “shows how” and “does” are expected [15] then other types of examinations such as the objective structured clinical examination (OSCE) are suggested [16]. Considering the positive correlation between knowledge and clinical performance, [17] we hope some changes may occur in participants’ clinical competence as the result of the knowledge they acquired.

Measurement of students’ satisfaction is another way to evaluate an online educational program [4]. In the current study, however, we have not measured students’ satisfaction with the program; rather, the willingness to complete this study reflected their perception regarding traditional and virtual learning methods. All students in the traditional group completed the study and three students in the experimental group expressed their willingness to attend the traditional sessions. Higher participation of the control group might have been attributed to the fact that students normally attend traditional lecture-based instruction, in which they feel more comfortable. Despite including computer literacy in our inclusion criteria, the unwillingness of three experimental group students to participate in the virtual learning environment might be due to their fear of using technology, and the possibility of failure in completing the course. In one study it was suggested that computer literacy among teachers and students was limited and should be enhanced [15].

Beside the necessity of preparedness of students for the use of online learning other factors may impact students’ preferences for online rather than in-class courses. Personality types, for example, have been highlighted by Daughenbaugh et al. According to these researchers, more extroverted students and those more sensitive than intuitive preferred the way the information was presented as well as the manner in which they were evaluated during online courses [18].

Although the experience of developing and using online learning was new in our dental school, the study results revealed that the program was feasible and had the better effectiveness.

The design of the current study was a post-test only design with the control group as a quasi-experimental design. Although these designs do not have extensive limitations, we propose to conduct further researches based on more rigorous designs such as experimental ones. In the current study the subject of teaching was practical, whereas the assessment was based on theoretical material. We recommend the assessment of practical ability in addition to the usual written tests. Our main limitation was the small number of participants. To generalize the findings and demonstrate more valid results, conducting this study with more participants is highly recommended.

## Conclusion

Despite the difficulties encountered in designing the virtual learning environment, the study was conducted successfully. Based on the findings of this study the virtual learning was more effective than lecture-based training.

## Competing interests

The authors declare that they have no competing interests.

## Authors’ contributions

MF Devised the study concept, designed the study, supervised the intervention, data collection and analysis, participated in the coordination of the study, and critically revised the manuscript. AMR collected data, ran the study intervention, participated in the study concept, performed the analyses, and drafted the manuscript. BE contributed to the design and analysis of the study data, and revised the manuscript. JF contributed to the design and intervention of the study, and manuscript revision. MM participated in the intervention, data collection and revision of the manuscript. All authors read and approved the final manuscript.

## Pre-publication history

The pre-publication history for this paper can be accessed here:

http://www.biomedcentral.com/1472-6920/14/45/prepub
